# Celastrol inhibits ezrin-mediated migration of hepatocellular carcinoma cells

**DOI:** 10.1038/s41598-020-68238-1

**Published:** 2020-07-09

**Authors:** Shihao Du, Xiaoyu Song, Yuan Li, Yalei Cao, Fuhao Chu, Olanrewaju Ayodeji Durojaye, Zeqi Su, Xiaoguang Shi, Jing Wang, Juan Cheng, Tangshun Wang, Xiang Gao, Yan Chen, Wuzhekai Zeng, Fengsong Wang, DongMei Wang, Xing Liu, Xia Ding

**Affiliations:** 1 School of Traditional Chinese Medicine, Beijing University of Chinese Medicine, Beijing, 100029 China; 2 Dongzhimen Hospital Affiliated to Beijing University of Chinese Medicine, Beijing, 100700 China; 3 MOE Key Laboratory of Membraneless Organelle and Cellular Dynamics, Hefei National Laboratory for Physical Sciences at the Microscale, University of Science and Technology of China, Hefei, 230027 China; 4 School of Life Science, Anhui Medical University, Hefei, 230032 China

**Keywords:** Cell migration, Liver cancer, Biochemistry, Chemical biology

## Abstract

Progression of hepatocellular carcinoma involves multiple genetic and epigenetic alterations that promote cancer invasion and metastasis. Our recent study revealed that hyperphosphorylation of ezrin promotes intrahepatic metastasis in vivo and cell migration in vitro. Celastrol is a natural product from traditional Chinese medicine which has been used in treating liver cancer. However, the mechanism of action underlying celastrol treatment was less clear. Here we show that ROCK2 is a novel target of celastrol and inhibition of ROCK2 suppresses elicited ezrin activation and liver cancer cell migration. Using cell monolayer wound healing, we carried out a phenotype-based screen of natural products and discovered the efficacy of celastrol in inhibiting cell migration. The molecular target of celastrol was identified as ROCK2 using celastrol affinity pull-down assay. Our molecular docking analyses indicated celastrol binds to the active site of ROCK2 kinase. Mechanistically, celastrol inhibits the ROCK2-mediated phosphorylation of ezrin at Thr567 which harnesses liver cancer cell migration. Our findings suggest that targeting ROCK2-ezrin signaling is a potential therapeutic niche for celastrol-based intervention of cancer progression in hepatocellular carcinoma.

## Introduction

Hepatocellular carcinoma (HCC) presents a major health threat worldwide, especially in South-East Asia. It ranks third among all malignancies both in incidence and mortality in China and accounts for approximately 42.5% of the total incidence worldwide^1,2^. Despite advances in its treatment, liver cancer remains the most difficult cancer to prevent. Surgical treatment of HCC is the first choice for patients to achieve long-term survival if HCC was diagnosed in the early stage. However, the recurrence rates after surgery were 50% at 3 years and 70% at 5 years^3^. Metastasis-associated recurrence is the major cause of poor prognosis in HCC. Although after years of continuous efforts, the long-term results of using existing therapies are still frustrating. Therefore, inhibiting the invasiveness of tumor cells is the key to improving the survival rate of cancer patients.

The members of the ezrin/radixin/moesin (ERM) family proteins are involved in multiple physiological and pathological phenomenon by acting as cross-linkers between actin cytoskeleton and plasma membrane in polarized cells^4–7^. As the founding member of the ERM, ezrin is essential for cell migration^8^, inflammatory response regulation^9^, villus morphogenesis^10^ and gastric acid secretion^11–14^. It has also been proved to be an important marker for cancer metastasis^15,16^. Our previous studies have shown that the phosphorylation of ezrin, such as the phosphorylation of ezrin in Thr567 and Ser66, is functionally related to membrane dynamics and plasticity^17–19^ and hyper-phosphorylation of ezrin at Thr567 is associated with HCC progression and metastasis^20^. The conserved threonine residue T567 of ezrin can be phosphorylated in a tissue dependent manner by ROCK in hepatocellular carcinoma^20^, MST4 in gastrointestinal epithelial cells^19,21^ and PKCα in breast cancer^22^.

ROCK kinase plays critical roles in tumor metastasis^23^ and ROCK inhibitors have shown promising effect on cancer cell migration^24^. ROCK and JAK1 signaling cooperate to control actomyosin contractility in carcinoma-associated fibroblasts to permit the melanoma cell migration^25^. According to our previous studies, ROCK hyper-phosphorylating on ezrin is related to liver cancer metastasis^20^. To date, a dominant view has been proposed that activation of ROCK by RhoA-GTP binding^26^ and caspase-mediated cleavage activation in apoptosis^27,28^.

Celastrol, isolated from medical plant Tripterygium wilfordii Hook F has multiple pharmacological activities, such as anti-cancer, anti-inflammation and anti-obesity^29,30^. Evidence has accumulated to indicate its potential in blocking tumor cell migration and angiogenesis^31^. In this study, we screened a series of TCM monomers that have been reported to have anti-cancer effect, to see its effect on liver cancer cell migration, finding that celastrol is the most powerful monomer in inhibiting liver cancer cell migration. However, the mechanism behind remains elusive. Therefore, we focused on the possible mechanism of celastrol inhibiting cancer cell migration, in order to provide research basis for the treatment of HCC.

In hepatocellular carcinoma cells, activation of ezrin is essential for cell migration and intrahepatic metastasis. Here we identified a novel traditional Chinese medicine monomer, celastrol, which disturbs the liver cancer cell migration. Celastrol prevents the ROCK kinase activity via binding to the catalytic subunit, but not the substrates binding affinity. Our results elucidate a novel chemical inhibitor of ROCK, and its mechanism on the ROCK kinase activity regulation.

## Results

### Identification of HCC migration inhibitors using natural products from Chinese herbs

Recent study unraveled the importance of ezrin protein acetylation in CCL18-elicited breast cancer metastasis^32^. Since our early study revealed the role of ezrin Thr567 phosphorylation in promoting intra-hepatocellular metastasis in xenografts and ezrin T567 phosphorylation in a context-dependent manner^20^, we sought to screen inhibitors to prevent the intra-hepatocellular carcinoma metastasis. In advantage of the abundant and available of traditional Chinese medicine monomers, we detect the inhibition of hepatocellular carcinoma cell migration in our monomer library via wound healing assay. According to our results, curcumin, celastrol, artesunate, dihydroartemisinin, berberine, astragaloside IV could inhibit hepatocellular carcinoma cell migration. Among them, celastrol dramatically suppresses the cell migration rate up to 40% compared to control in wound healing assay (Figure S1A).

### Cytotoxic effect of celastrol on hepatocellular carcinoma cells

To precisely evaluate the effects of celastrol on the cancer cells, the cytotoxic effect of celastrol on the viability of HepG2 and MHCC97H cells was determined by MTS assay. HepG2 and MHCC97H cells were treated with different concentrations of celastrol (0 nM, 5 nM, 10 nM, 50 nM, 100 nM, 500 nM, 1 μM, 5 μM, 10 μM) for 24 h before MTS assay. Compared with the control (0.1% DMSO), cell viability was significantly inhibited above 1 μM for both MHCC97H and HepG2 cell lines (Figure S1B, C, *P < 0.05, **P < 0.01). As the high concentration of celastrol shows cytotoxic effect and caused serious side effect in vivo, we would like to select a relative low concentration of celastrol, which could inhibit the migration of hepatocellular carcinoma cells.

### Celastrol inhibits HCC cell migration

**Figure 1:**
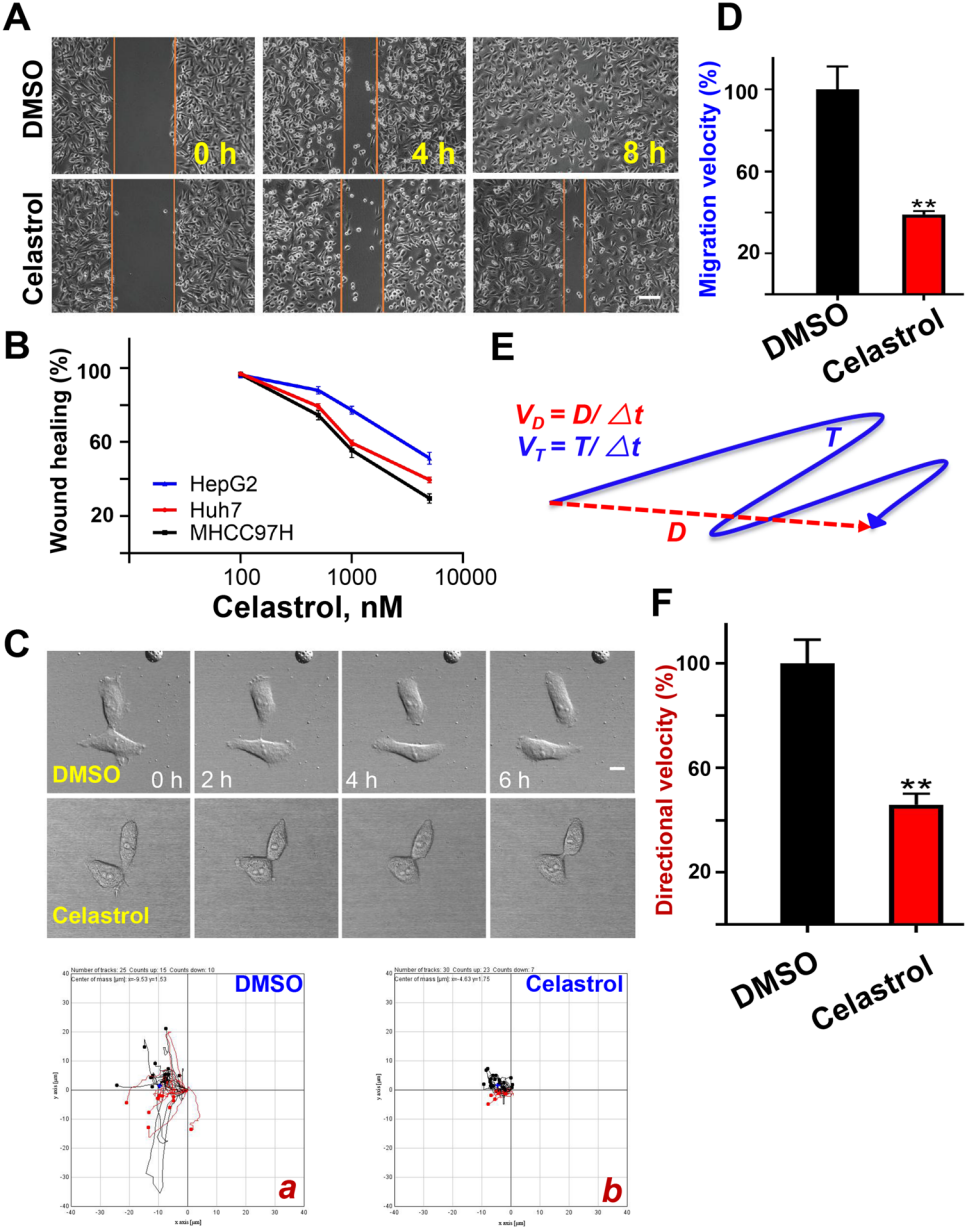
Celastrol inhibited hepatocellular carcinoma cell migration. (**A**) Wound healing area of MHCC97H cell. Images were collected before or 4 and 8 h after 20% fetal calf serum addition. 2‰ DMSO were added to the Control group and 500 nM celastrol were added to the treatment group. Bar = 100 μm. (**B**) Quantitative analyses of wound-healing assay. MHCC97H, Huh7 and HepG2 cells were treated with different concentration of celastrol for 8 h. The wound healing percentage were calculated by dividing the wound healing area of each by that of control respectively. (**C**) Single cell tracking of MHCC97H cells. MHCC97H cells were treated with 500 nM celastrol (**b**) or equal volume of DMSO (**a**) and then imaged at every 10 min for 6 h. The migration track of total cells was shown in (**a**, **b**). Bar = 50 μm. (**D**) Quantitative analysis of the relative migration velocity. Migration velocity of cells treated with 500 nM celastrol and control were calculated. The rate was calculated by comparing the velocity with control group and expressed as percentage. *P < 0.05, **P < 0.01. (**E**) Diagram of migration path length. The total distance between starting and ending points (T) and the actual trajectory (D) are indicated. Migration velocity (υT) and directional migration velocity (υD) were calculated as illustrated. υT is the ratio of total track distance to total time, and υD is the ratio of direct distance between the start and end point to total time. (**F**) Quantitative analysis of the relative directional velocity. Directional velocity of cells treated with 500 nM celastrol and control were calculated

In order to find the best concentration in inhibiting liver cancer cell migration, the gradient wound healing assays were detected on celastrol. As shown in Fig. 1A, the migration area of MHCC97H cells within 8 h was significantly decreased by celastrol treatment compared to the control group. To confirm whether the cellular response to celastrol is cell line oriented, we carried out the wound healing assay on another two hepatoma cell lines (Figure S2). As shown in Fig. 1B, the cell line related sensitivity as the IC50 values (2.94 μM for MHCC97H, 3.72 μM for Huh7, and 5.24 μM for HepG2) are different in those two cell lines. Since MHCC97H is a highly invasive cell line and sensitive to celastrol treatment, it was used for the following studies.

To confirm the celastrol inhibition on MHCC97H cell migration, the single cell tracking assay was carried out. Similar to the result of wound healing assay, compared to the control group, the cell migration track of celastrol treated MHCC97H showed dramatically inhibition (Fig. 1C). To quantitatively evaluate the celastrol effects on cell migration, the migration velocity (υT) and directional migration velocity (υD) of MHCC97H cells were calculated as done previously^32,33^. υT representing the cell migration velocity, while υD representing the directional migration velocity (Fig. 1E). In accordance to the cell migration tracks, the υT and υD was dramatically reduced in celastrol treated cells (Fig. 1D, F). Thus, we conclude that celastrol could effectually inhibit the migration of MHCC97H cell migration.

### ROCK is a potential target of celastrol in hepatocellular carcinoma cells

**Figure 2:**
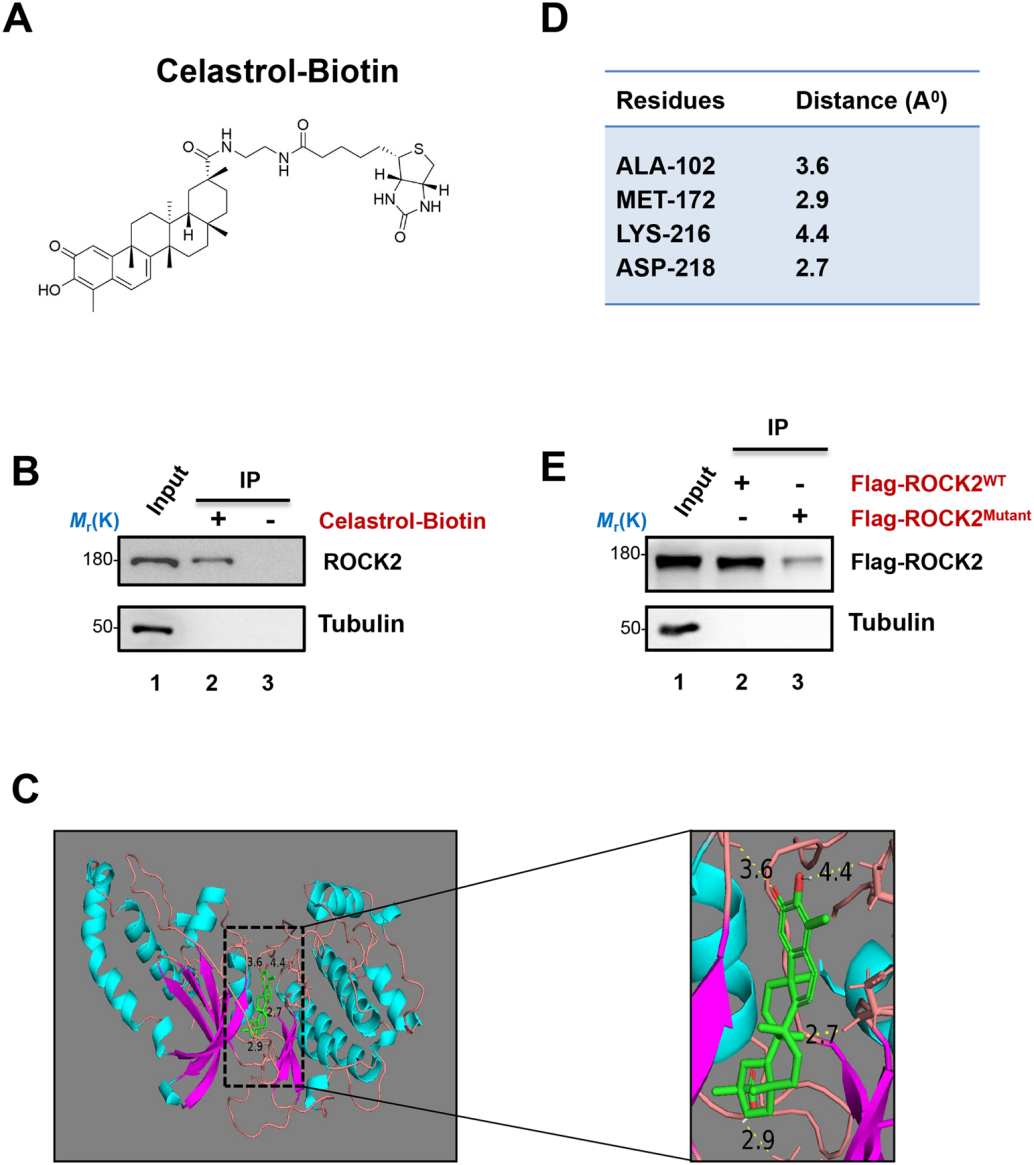
ROCK2 is a potential target of celastrol in hepatocellular carcinoma cells. (**A**) The molecular structure of celastrol biotin. (**B**) The western-blotting analyses of protein pulldown by celastrol-biotin. Celastrol-biotin were incubated in MHCC97H cell lysis and then immunoprecipitated with avidin beads. Full-length blots are presented in Supplementary Figure S8. (**C**) Cartoon representation of celastrol (green) docked onto the ROCK2 model. Potential area of celastrol interacted with the residues of ROCK2 were shown in yellow dotted lines. The numbers represent the distance between each residue and celastrol. (**D**) The amino acid residues of ROCK2 that formed polar bonds with celastrol and the distance between each residue and celastrol. (**E**) The western-blotting analyses of Flag-ROCK2 pulldown by celastrol-biotin. The ROCK2^WT^ represents MHCC97H transfected with Flag-ROCK2. The ROCK2^Mutant^ represents MHCC97H transfected with Flag-ROCK2 mutant (ALA-172, ALA-216 and ALA218). Full-length blots are presented in Supplementary Figure S8.

To resolve the mechanism of celastrol inhibition on the hepatocellular carcinoma cells migration, the potential targets need to be analyzed. Based on previous proteomic studies, ROCK was reported as a potential target of celastrol in lymphoblastic cells^34^. Our previous studies reveal that ezrin Thr567 phosphorylation plays an important role in the regulation of tumor metastasis in hepatocellular carcinoma and cellular polarity in polarized gastric cells^18–20^. Therefore, we would like to figure out whether celastrol inhibits cell migration via ROCK-ezrin phosphorylation signaling pathway. To this end, we labeled the celastrol by biotin via chemical reaction (Fig. 2A, S3A), the celastrol-biotin was further purified and detected by mass spectrum (Figure S3B). Then the celastrol-biotin was used for the biotin-affinity pulldown assay, the result showed that celastrol could bind to ROCK2 (Fig. 2B, S4A). To pinpoint the binding sites of celastrol with ROCK2, the computational molecular docking analysis was conducted. As showed in the modeling, celastrol could interact with the amino acid residues on the catalytic sites of ROCK2 (Fig. 2C, D) mainly comparable to the known ROCK 1 inhibitor Y-27632 (Figure S5). Common residues were observed to have interacted with both celastrol and Y27632. Celastrol which exhibited the best binding score was also observed to have formed polar bonds with four ROCK 2 residues, comparing to the three observed in the interaction with Y27632. That confirms the link between the abundance of polar bonds and the activity of inhibitors, whereas celastrol does not affect the substrate binding affinity (Figure S4B). In order to confirm the potential binding sites, we mutated the relevant site of ROCK2 into ALA-172, ALA-216 and ALA-218, and again carried out the biotin-affinity pulldown assay. As shown in Fig. 2E, the binding force of celastrol with ROCK2 mutant was reduced.

### Celastrol directly inhibits the kinase activity of ROCK

**Figure 3:**
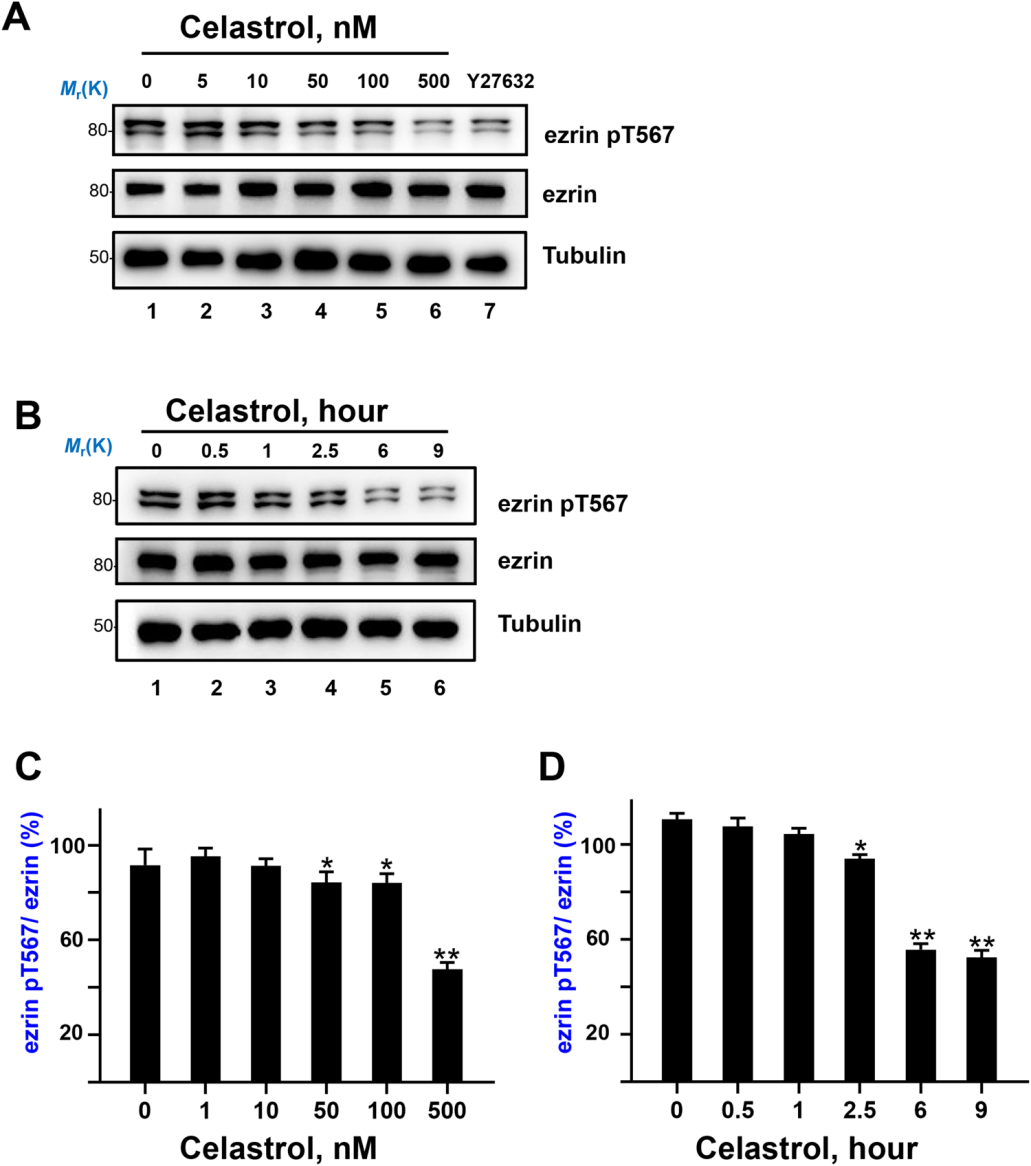
Celastrol inhibit the phosphorylation of ezrin Thr567 in a concentration and time dependent manner. (**A**) Western blotting analyses of ezrin pT567, ezrin and α-Tubulin, after treating MHCC97H cells with different concentration of celastrol for 6 h (Y27632 as a positive control). Full-length blots are presented in Supplementary Figure S8. (**B**) Western blotting analyses of ezrin pT567, ezrin and Tubulin, after treating MHCC97H cells with 500 nM celastrol for different time. Full-length blots are presented in Supplementary Figure S8. (**C**) Quantitative analysis of the density of the western-blotting in (**A**). Ratio was calculated by dividing the density of ezrin pT567 by that of ezrin, *P < 0.05, **P < 0.01. (**D**) Quantitative analysis of the density of the western-blotting in (**B**). Ratio was calculated by dividing the density of ezrin pT567 by that of ezrin, *P < 0.05, **P < 0.01.

According to the fact that ROCK2 serves as the potential target of celastrol, we next examined whether celastrol could inhibit the ROCK2 mediated ezrin Thr567 phosphorylation in hepatocellular carcinoma cells. To pinpoint the inhibition of celastrol, we treated the hepatocellular carcinoma cells in a concentration gradient to evaluate the ezrin phosphorylation. As shown in Fig. 3A, C, ezrin pT567 were significantly reduced with celastrol treatment compared to control or the lower concentration celastrol treatment, the inhibitory effect was similar to Y27632 (ROCK inhibitor). To confirm that the celastrol inhibition was not cell line-oriented, we carried out the detection in another two hepatocellular carcinoma cell lines, HepG2 and Huh7. Similar to the inhibition on MHCC97H cells, celastrol treatment inhibits ezrin Thr567 phosphorylation in Huh7 (Figure S6A) and HepG2 cells (Figure S6B) in a concentration-dependent manner. As ROCK2 and ezrin are compartmentalized in cells and accessibility of the celastrol to its target and dephosphorylation of pre-phosphorylated ezrin could be the time-limited factor, we then treated the hepatocellular carcinoma cells with 500 nM celastrol in time gradient. As shown in Fig. 3B, D, the ezrin Thr567 phosphorylation inhibition showed a time-dependent manner. Thus, we conclude that celastrol could inhibit the ROCK-mediated ezrin Thr567 phosphorylation in concentration- and time-dependent manners.

**Figure 4:**
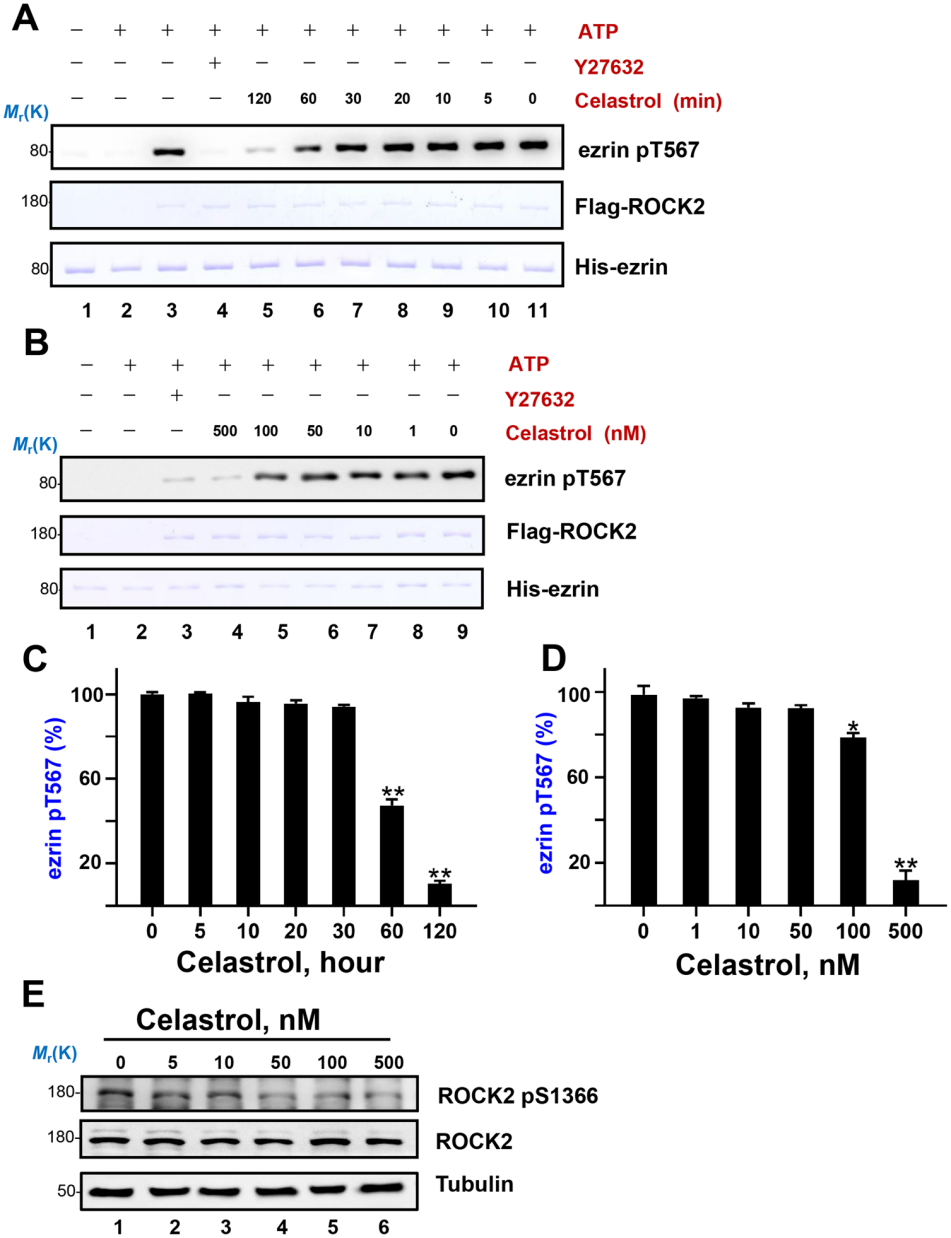
Celastrol inhibits the kinase activity of ROCK2. (**A**) The ROCK2 and ezrin were purified and subjected to in vitro phosphorylation in the presence of 500 nM celastrol for different time course (2 h, 1 h, 30 min, 20 min, 10 min, 5 min, 0 min) and Y-27632 (10 μM, 2 h). The phosphorylation level of ezrin Thr567 were measured by western-blotting. Full-length blots/gels are presented in Supplementary Figure S8. (**B**) The ROCK2 and ezrin were purified and subjected to in vitro phosphorylation in the presence of different concentration of celastrol (500 nM, 100 nM, 50 nM, 10 nM, 1 nM, 0 nM) and Y-27632 (ROCK inhibitor, 10 μM) for 2 h. The phosphorylation level of ezrin Thr567 were measured by western-blotting. Full-length blots/gels are presented in Supplementary Figure S8. (**C**) Quantitative analysis of the density of the western-blotting in (**A**). Ratio was calculated by dividing the density of different time course by that of 0 min. Significance were tested by multiple comparison with the density of 0 min group, *P < 0.05, **P < 0.01. (**D**) Quantitative analysis of the density of the western-blotting in (**B**). Ratio was calculated by dividing the density of different time course by that of 0 nM. Significance were tested by multiple comparison with the density of 0 nM group, *P < 0.05, **P < 0.01. (**E**) Western blotting analyses of ROCK2 pS1366, ROCK2 and α-Tubulin, after treating with different concentration of celastrol for 6 h. Full-length blots are presented in Supplementary Figure S8.

According to our computational molecular docking analyses, celastrol could inhibit the kinase activity of ROCK2 directly. To evaluate the direct inhibition effects, we purified FLAG-ROCK2 from HEK-293T cells and His-ezrin wild type from bacteria. In vitro phosphorylation assay was conducted with 500 nM celastrol in time gradient, the result indicated that celastrol inhibited ROCK2 phosphorylation on ezrin in a time dependent manner (Fig. 4A, C). We then carried out the phosphorylation assay with different concentration of celastrol treatment for two hours, the results revealed a concentration-dependent inhibition on ezrin Thr567 phosphorylation in vitro (Fig. 4B, D) as cellular experiments. As reported, ROCK2 kinase activity was regulated by increasing its auto-phosphorylation on ROCK2 Ser1366^35^. To find out the relationship between celastrol and ROCK2 kinase activity in hepatocellular carcinoma cells, ROCK2 Ser1366 phosphorylation was detected in celastrol treated cells. In a line with our ezrin Thr567 phosphorylation inhibition results, celastrol inhibits the phosphorylation of ROCK2, and therefore perturbs its kinase activity in hepatocellular carcinoma cells (Fig. 4E). Together with our previous ROCK-ezrin signaling regulates the intrahepatic cancer metastasis, we identified a novel chemical drug, celastrol, which could target the ROCK2-ezrin signaling as a potential therapeutic drug to prevent the hepatocellular carcinoma metastasis. We then investigated the genomic characteristic of hepatocellular carcinoma in TCGA database, finding that ROCK2 was altered in 30 samples out of 371 (Figure S7). Therefore, celastrol might be a strategy for those patients.

### Celastrol inhibits HCC cell migration via ROCK2 mediated ezrin T567 phosphorylation

**Figure 5:**
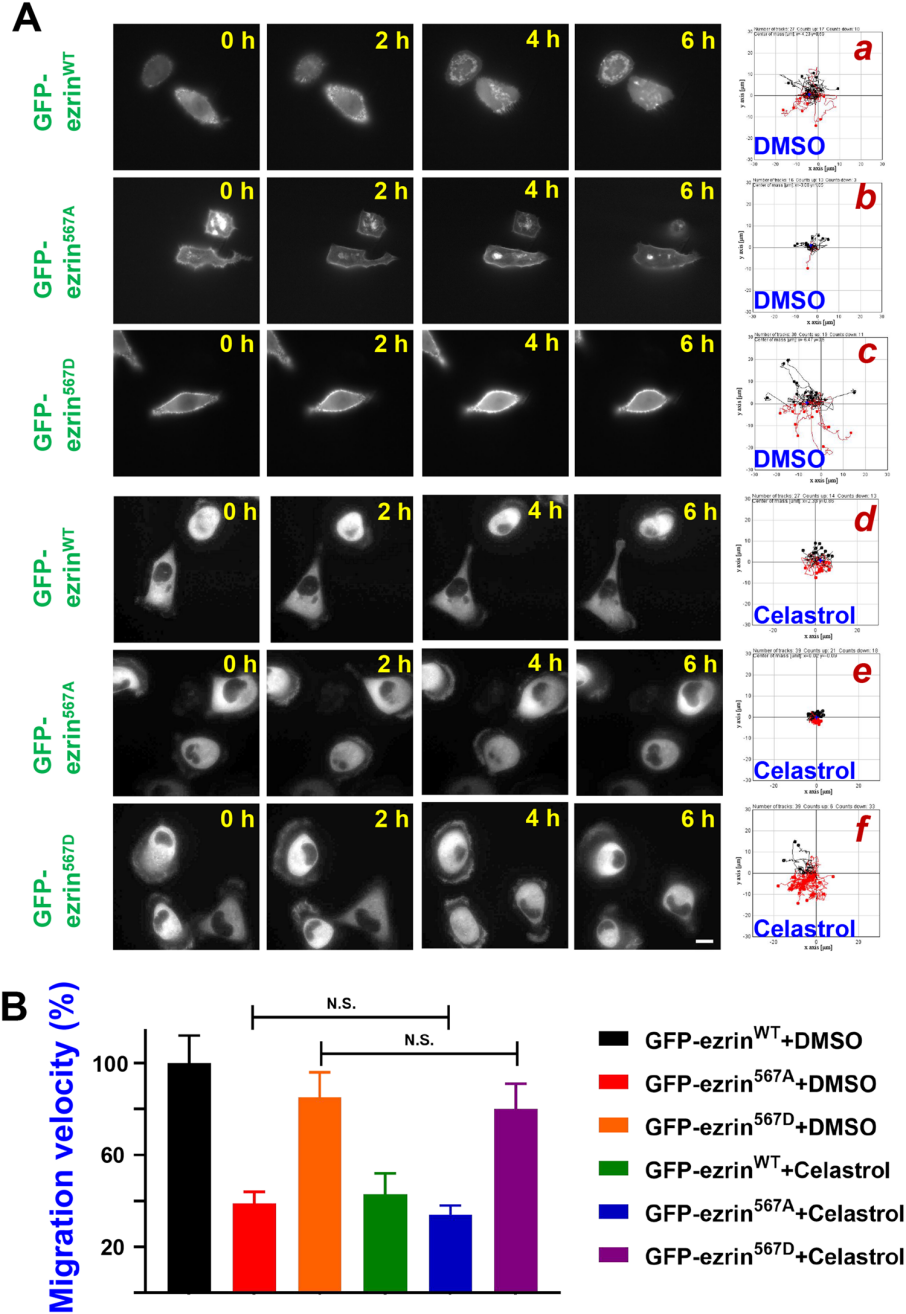
Celastrol inhibits HCC cell migration via ROCK2 mediated Ezrin T567 phosphorylation. (**A**) Single cell tracking of MHCC97H cell transfected with ezrin mutant were treated with 500 nM celastrol or control. MHCC97H transfected with pEGFP-ezrin^WT^, pEGFP-ezrin^567A^ or pEGFP-ezrin^567D^ were treat with 500 nM celastrol (**d**, **e**, **f**) or equal volume of DMSO (**a**, **b**, **c**) and then imaged at every 10 min for 6 h. The migration track of total cells was shown in (**a**, **b**, **c**, **d**, **e**, **f**). Bar = 50 μm. (**B**) Quantitative analysis of the relative migration velocity. The rate was calculated by comparing the velocity with control group (pEGFP-ezrin^WT^ with DMSO treatment) and expressed as percentage. N.S. represents *P* > 0.05.

It has been proved that ROCK2 knockout and Thr567 nonphosphorylation mutant could efficiently reduce hepatoma cell velocity^20,36^. In order to verify celastrol’s inhibitory effect on ROCK2 phosphorylating ezrin Thr567, we transfected MHCC97H with pEGFP-ezrin^567D^ (phospho-mimicking), pEGFP-ezrin^567A^ (nonphospho-mimicking) or pEGFP-ezrin^WT^ plasmids. The single cell tracking assay was carried out on each group to analyze the velocity of control and celastrol treatment. As shown in Fig. 5A, the celastrol treatment has the similar effect as that of ROCK2-nonphospho-mimicking ezrin mutant transfection. Importantly, this stimulated HCC cell migration was not inhibited by celastrol, indicating that ROCK2-ezrin signaling drives HCC cell migration and inhibition of ROCK2 is sufficient to attenuate the migration (Fig. 5B). This experiment further confirmed our hypothesis, which celastrol inhibits hepatoma cell migration via ROCK2-ezrin pathway.

## Discussion

HCC is one of the most common global malignancies due to its invasive phenotype of intra-hepatic metastasis. We show that ROCK2 is a novel target of celastrol and inhibition of ROCK2 suppresses elicited ezrin activation and liver cancer cell migration. Our molecular docking analyses and biochemical characterization indicated celastrol binds to the active site of ROCK2 kinase and celastrol binding minimized ROCK2-mediated phosphorylation of ezrin at Thr567 which harnesses HCC cell migration and invasion. Our findings suggest that ROCK2-ezrin interaction is a useful niche for interrogation of HCC progression.

Celastrol was one of the most promising natural products to be deployed in the clinic^37^. According to the present stage of research, celastrol has shown inhibitory effect on cancer initiation^38^, proliferation^39^, angiogenesis^40^, drug-resistance^41^ and metastasis^42^. In this study, we found that celastrol dramatically inhibited liver cancer cell migration in vitro which is likely the mechanism of action underlying celastrol-mediated inhibition of hepatocellular carcinoma invasiveness in vivo. According to previous studies, celastrol takes anti-metastasis effect via multiple targets, such as inhibiting PTEN/PI3K/AKT pathway^43^, NF-κB pathway^44^ and HSP90-HIF1α-VEGF pathway^45^. Moreover, celastrol could also target tumor microenvironment, such as inhibiting IL-1β production^30^, suppressing M2-like polarization macrophages^42^, inhibiting angiogenesis^40^ and reducing matrix metalloproteinase^46^. In this study we found that the inhibitory effect on hepatoma cell migration was taken through ROCK2-ezrin pathway. However, the side effect of high concentration celastrol, such as cardiotoxicity and reproductive toxicity, could not be neglected^47–50^. Both hepatocytes and hepatoma cells viability were significantly inhibited by high concentration celastrol treatment^51^. Therefore, our wound-healing experiment was done in a narrow time-window, this protocol had precluded the effect of proliferation. Since the binding affinity of celastrol to ROCK2 is confirmed, the SAR optimization^52^ is needed for translational application of celastrol. Currently, we are establishing hepatocyte organoids model so we can model the HCC progression and screen for potent compounds derived from celastrol.

ROCK kinase was first identified as a GTP-RhoA effector^53^, with an important role in actin organization and cell migration. The aberrant regulation of ROCK is related to multiple diseases, such as cancer, cardiovascular disease^54^ and neurological disorders^55^. ROCK kinase family, ROCK1 and ROCK2, is consisted of two members with shared kinase domain at N-terminal, followed by a central coiled to coil region, a PH domain and a CRD domain at C-terminal. During inactive state, the C-terminal act as an auto-inhibitory region. Several lines of evidence indicate that ROCK2, instead of ROCK1 is overexpressed in hepatocellular carcinoma^36^. Interestingly, it has also been reported that ROCK2, instead of ROCK1 were disturbed by celastrol^56^. In this study, the binding affinity between ROCK2 and celastrol were predicted by molecular docking and confirmed by celastrol-biotin affinity pulldown assay. Moreover, the in vitro phosphorylation assay indicates that celastrol perturbs ROCK2 kinase activity on its substrate ezrin.

Ezrin was first identified as an actin binding protein, which serves as a cross linker of membrane and cytoskeleton^12^. It consists of conservative FERM domain in the N terminal, α-helix, linker region and auto inhibited ERMAD domain in the C terminal^57^. The phosphorylation of Thr567 will reduce the affinity of the C-ERMAD for FERM domain, and thus induce the formation of F-actin-membrane linkages. The conserved threonine residue Thr567 of ezrin can be phosphorylated in a tissue- and cell-dependent manner. For example, PKC in breast cancer cell^22^ and MST4 in gastrointestinal epithelial cells^19^ phosphorylates ezrin Thr567. Hyper phosphorylation of ezrinThr567 by ROCK is responsible for hepatocellular cell invasiveness^20^.

Recent studies showed that ezrin and its interaction partner ACAP4 acetylation is involved in breast cancer metastasis in response to cytokine CCL18 stimulation^32,58^. Given the hyper-activation of Aurora kinase activity in HCC^59^, acetyl-phosphorylation cross-talk on the regulation of cellular dynamics^60^, it would be of great interest to delineate the spatiotemporal dynamics of ezrin acetylation and ROCK2-mediated phosphorylation in HCC metastasis. Interestingly, ACAP4 was initially identified as a transcript highly expressed in liver cells^17,18^. Our future studies will generate human hepatocyte organoids from patients with high and low ACAP4 levels and test whether celastrol exhibits any selectivity in the context of ACAP4 activity and identify if there are additional effectors downstream from ROCK2 in HCC cell migration.

In sum, this study demonstrated that celastrol significantly inhibits liver cancer cell migration in vitro. The mechanism behind might be that celastrol disturb the kinase domain of ROCK2 by perturbing its catalytic site, and thus decreases the phosphorylation level of ezrin Thr567. The results provided the supports on the use of celastrol as a potential clinical intervention for preventing and delaying liver cancer metastasis. Therefore, celastrol treating hepatocellular carcinoma warrants further preclinical investigation.

## Materials and methods

### Chemicals and antibody

Celastrol was ordered from MedChemExpress under the item number HY-13067. Antibodies against ezrin, ezrin pT567 and α-tubulin (DM1A) were purchased from Cell Signaling Technology. Antibodies against ROCK2 and ROCK2 (phospho S1366) were purchased from Abcam. Anti-GFP antibodies were purchased from Santa Cruz Biotechnology. Anti-FLAG-tag (M2) antibody was from Sigma.

### Plasmids

The development of bacterial expression vectors containing human ezrin fused to histidine was described previously^61^. EGFP-tagged ezrin plasmids was produced as described before^62^. PCR amplified ROCK2 cDNA was cloned into the 3× FLAG-Myc-CMV-24 vector (Sigma) by *BamH*I and *RcoR*I digestion. The mutanted of ezrin and ROCK2 were constructed by Fast Mutagenesis Kit (Vazyme Biotech). All plasmids were verified by sequencing (Tsingke Biological Tech).

### Cell culture and transfection

MHCC97H was a gift from Professor Yong Chen (Fourth Military Medical University)^20^. HepG2 and HEK293T cells were from American Type Culture Collection (ATCC). Huh7 cells were from National Infrastructure of Cell Line Resource. The cells were incubated according to ATCC instruction. Cells were transfected using conventional calcium phosphate method or the Lipofectamine 3,000 (Invitrogen) according to manufacturer’s instructions.

### Determination of cell viability (MTS assay)

Celastrol cytotoxicity was assessed with the use of a CellTiter 96 AQueous One Solution Cell Proliferation Assay (Promega), which is a form of the 3-(4,5-dimethylthiazol-2-yl)-5-(3-carboxymethoxyphenyl)-2-(4-sulfophenyl)-2*H*-tetrazolium inner salt (MTS) assay. Cells (5,000 cells/well) were plated in 96-well plates, treated with various concentrations of celastrol for 24 h. After celastrol treatment, cell viability was determined by adding a small amount of the One Solution Reagent directly to culture wells, incubating for 3 h and then recording the absorbance at 490 nm with a 96-well plate reader.

### Wound healing assay

MHCC97H, HepG2 or Huh7 cells with logarithmic growth phases, was cultivated in 12 orifices with 5 × 10^5^ cells density. Each group has three replicates. After a starvation of 6 h in serum-free medium, 10 μl Tip was taken to vertical scratch "#" glyph at the bottom of the culture plate. The floating cells were washed off with PBS and the celastrol was dissolved in medium supplemented with 20% FBS. Samples were photographed at 0 h, 4 h and 8 h. Image J software was used to measure and compare the effects of celastrol on cell migration.

### Single cell tracking assay

The cell migration by single cell tracking assay was performed as previously described^32^. Briefly, MHCC97H cells were laid in a Silian dish. After stabilized, the cells were starved with Opti-MEM for 6 h, and then cultured in DMEM containing 20% FBS. The movement track of 10 single-cells in each field of view were recorded by microscope for 6 h, with 10 min interval at once.

### Western blotting

Protein samples were collected and equivalent aliquots of protein were electrophoresed on a 10% SDS/polyacrylamide gel in 1 × Tris-glycin buffer at first, and then transferred to nitrocellulose membranes in 1 × Trans Buffer, finally incubated with primary antibodies overnight at 4 °C. Following incubation with secondary antibody 1 h at room temperature, the immunoreactive proteins were detected by enhanced Chemo luminescence Substrate, and the blot was scanned and densitometric analysis with Image J software.

### Celastrol-biotin affinity pulldown assay

The biotin-affinity pulldown assay was performed as previously described^63,64^. Briefly, Celastrol-biotin were synthesized and verified by the mass spectrometry. MHCC97H were lysed in lysis buffer (50 mM Tris, pH 7.4, 150 mM NaCl, 0.1% (vol/vol) Triton X-100, 5 mM EDTA and protease inhibitors) and centrifuged at 16,000×*g* for 20 min at 4 °C. Then the cell lysates were incubated with biotin-celastrol (10 μM) or DMSO for 2 h. The supernatant was incubated with Avidin Agarose (Thermo Fisher Scientific) for 4 h. The beads were washed three times and then incubated in lysis buffer with 10 µM celastrol for 30 min to compete with biotin-celastrol. Then the supernatant was removed and the beads were boiled in SDS-PAGE sample buffer. The sample were test by immunoblot with indicated antibody.

### Recombinant proteins purification

His-ezrin proteins were produced in Rosetta (DE3). Briefly, the plasmids were transformed into E. coli strain, and protein expression was induced with 1 mM IPTG at 16 °C for 20 h. Then the protein was purified using Ni-NTA agarose (Qiagen) according the manufacturer’s instructions. Flag-ROCK2 were transfected into HEK293T cells and then enriched by FLAG-M2 resin (Sigma). All purification procedures were performed at 4 °C, and protease inhibitor cocktail (Sigma) was added to prevent protein degradation. All purified proteins were analyzed and confirmed with SDS/PAGE.

### In vitro phosphorylation assay

The kinase reactions were performed in 40 μl of 1 × kinase buffer (25 mM HEPES, pH7.2, 50 mM NaCl, 2 mM EGTA, 5 mM MgSO_4_, 1 mM DTT, and 0.01% Brij35), recombinant ezrin proteins (2 μg) as substrate and recombinant ROCK2 (100 ng) as kinases. Reaction mixtures were incubated at 30 °C for indicated times, then terminated by 5 × SDS-PAGE loading buffer (10% SDS; 0.5% bromophenol blue; 50% glycerol; 100 mM DTT). After the samples were boiled at 100 °C for 2 min, 50% of the sample was resolved by SDS-PAGE and stained by Coomassie Brilliant Blue R250.

### Molecular docking

The ROCK2 amino acid sequence was retrieved from the National Center for Biotechnology Information (NCBI) Database. The 2D structure of celastrol and the Y27632 (ROCK1 inhibitor) were designed using the Marvin Sketch software. Weak interactions between celastrol and ROCK2 were visualized using the PyMOL molecular graphics system. The binding energy scores between the experimental ligands and ROCK2 was predicted using the Auto Dock Vina software.

### Measurement of ROCK2 mRNA levels

The mRNA expression data of ROCK2 were extracted from TCGA datasets within cBioPortal (https://www.cbioportal.org/)^65,66^. OncoPrint, a graphical summary of genomic alterations, gives an overview of genomic alterations to ROCK2 in a selected cohort. Clinical attributes can also be visualized together with the genomics data.

### Statistical analysis

The GraphPad Software was used to make statistical graph. The SPSS 20.0 software was used for statistical analysis. Data shown were either representative of three or more independent experiments. Data was analyzed as mean ± SE. Student’s t test and one-way ANOVA were used for statistical analysis. Difference was considered significant when the two-sided p-value was less than 0.01.

## Supplementary information

Supplementary Information.
