# Supplementary material for: Celastrol inhibits ezrin-mediated migration of hepatocellular carcinoma cells
Source: Sci Rep. 2020 Jul 9;10:11273. doi: 10.1038/s41598-020-68238-1 (PMC7347585; doi:10.1038/s41598-020-68238-1)

Celastrol inhibits ezrin-mediated migration of hepatocellular carcinoma cells

Shihao Du[1, 2, 5], Xiaoyu Song[3, 5], Yuan Li[2, 5], Yalei Cao[1,5], Fuhao Chu[1], Olanrewaju Ayodeji Durojaye[3], Zeqi Su[1], Xiaoguang Shi[2], Jing Wang[2], Juan Cheng[2], Tangshun Wang[2], Xiang Gao[2], Yan Chen[1,2], Wuzhekai Zeng[3], Fengsong Wang[4], DongMei Wang[3], Xing Liu[3], Xia Ding[1,2,*]

1 School of Traditional Chinese Medicine, Beijing University of Chinese Medicine, Beijing, China 100029; 2 Dongzhimen Hospital Affiliated to Beijing University of Chinese Medicine, Beijing, China 100700; 3 MOE Key Laboratory for Membraneless Organelles & Cellular Dynamics & Hefei National Center for Physical Sciences at the Microscale, University of Science & Technology of China, Hefei, China 230027; 4 School of Life Science, Anhui Medical University, Hefei, China 230032.

5 Equal contribution

*Correspondence: dingx@bucm.edu.cn

Supplemental Figure 1

Du *et al*.,

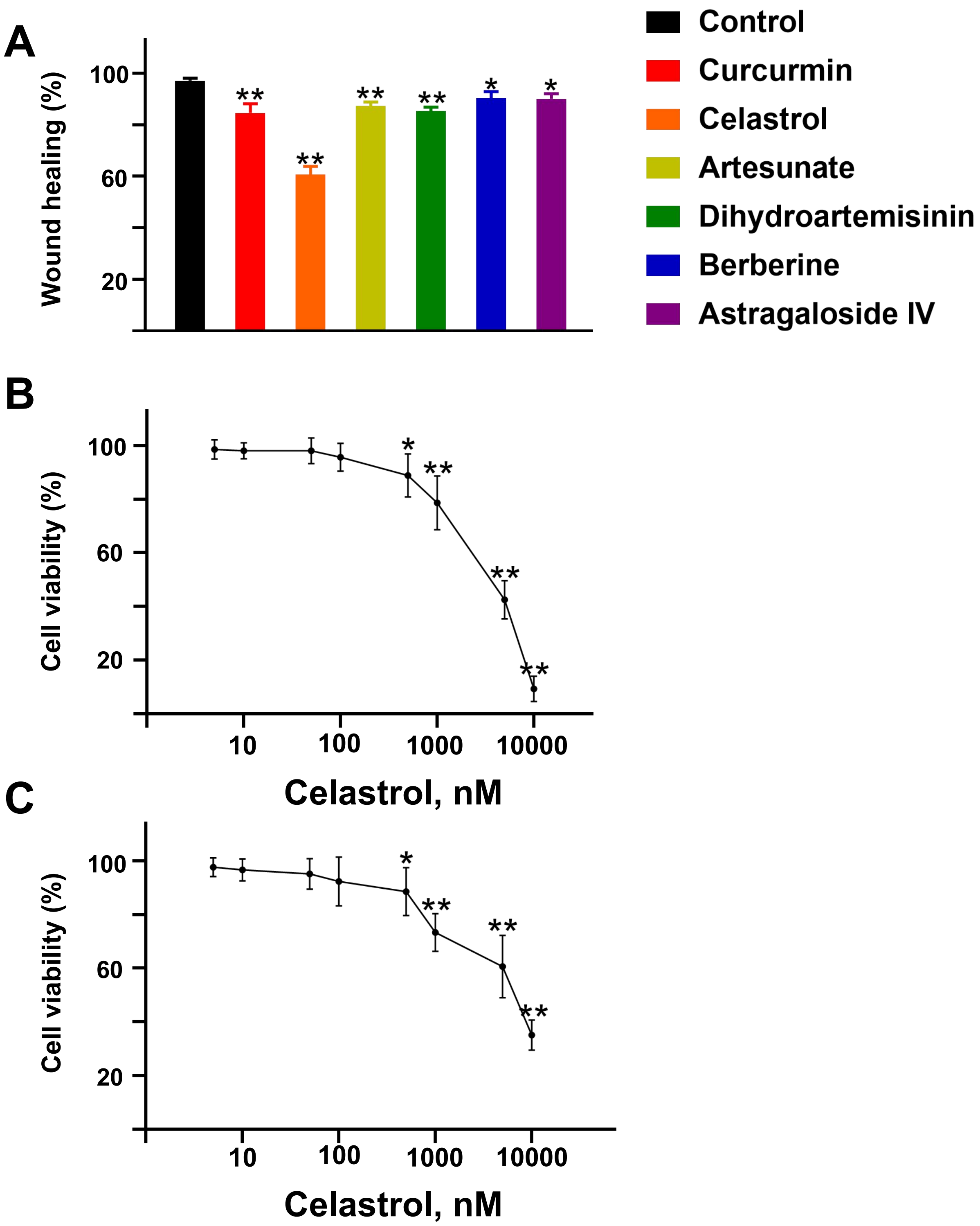

Figure S1

A. The inhibitory effect of several TCM monomers (1μM) on MHCC97H cell motility were tested by wound healing assay. The wound healing ratio (%) of 8 hours were calculated.
B. MHCC97H cells were treated with 2‰ DMSO and different concentration of celastrol (5 nM, 10 nM, 50 nM, 100 nM, 500 nM, 1 μM, 5 μM, 10 μM) for 24h before MTS assay, the cell viability was calculated by dividing the OD of each group by OD of the control group.
C. HepG2 cell were treated in the same way as above. The *error bars* represent S.E.; *n* = 3 preparations, *P<0.05, **P < 0.01.

Supplemental Figure 2

Du *et al.*,

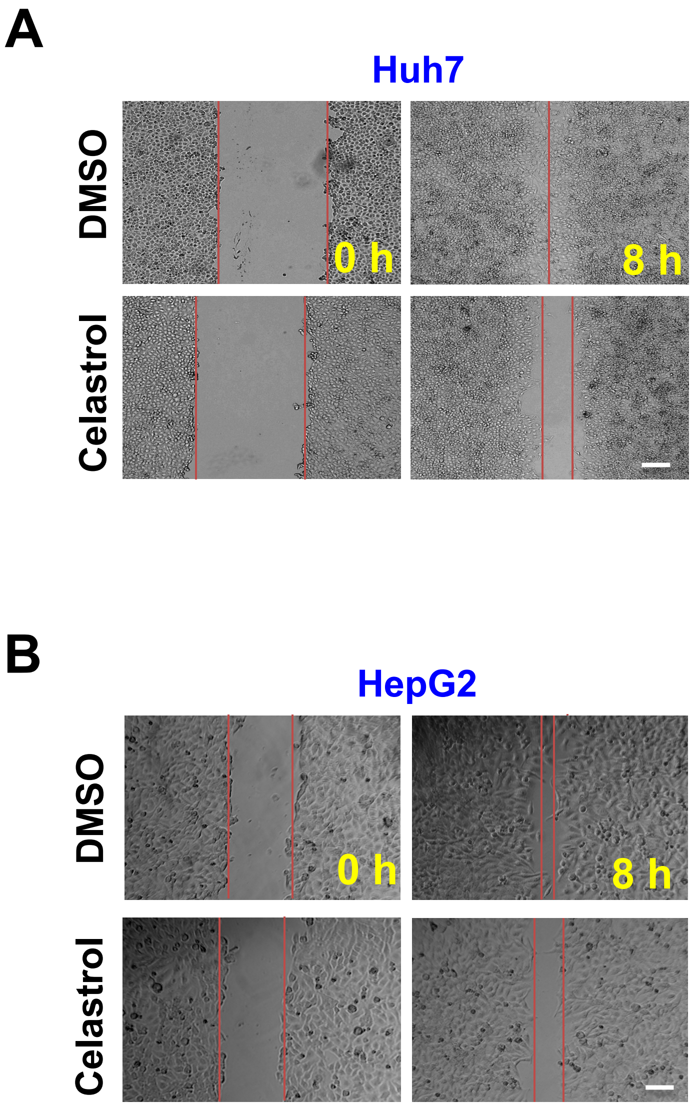

Figure S2

A. Wound healing area of Huh7 cell. Images were collected before and 8 hours after 20% fetal calf serum addition. 2‰ DMSO were added to the Control group and 500 nM celastrol were added to the treatment group. Bar = 100 μm.
B. Wound healing area of HepG2 cell.

Supplemental Figure 3

Du *et al*.,

A

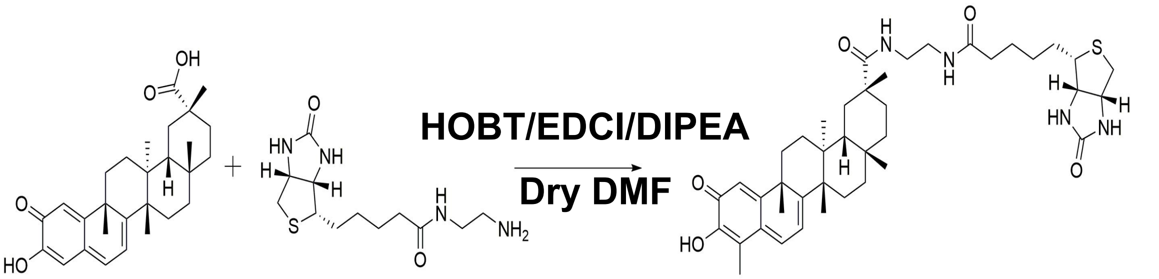

B

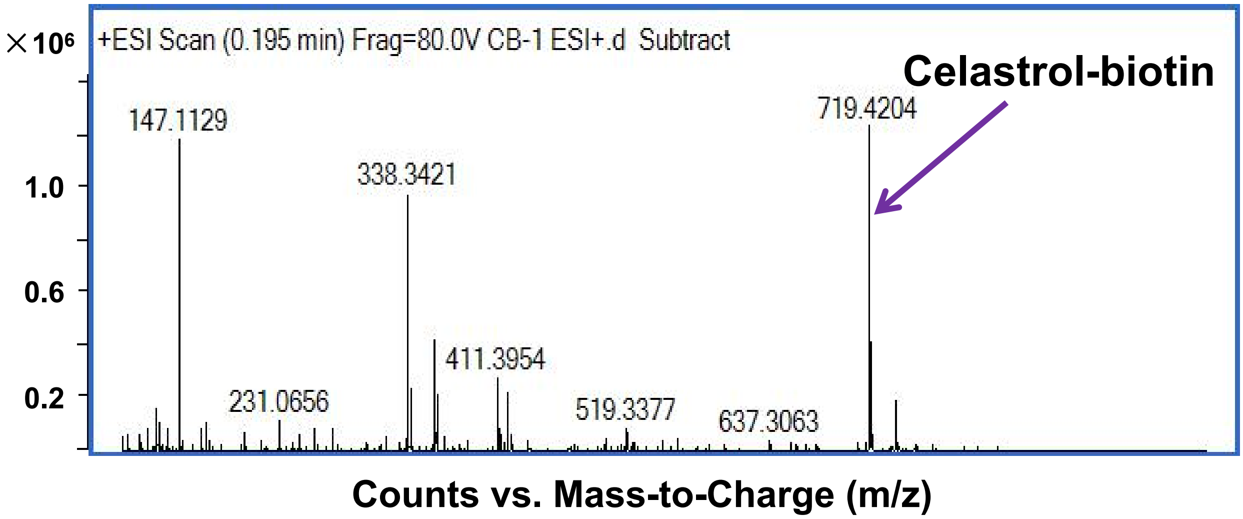

Figure S3

A. The flowchart shows the synthesis process of celastrol-biotin.

B. The mass spectrometry identified the celastrol-biotin (peak 719.4204).

Supplemental Figure 4

Du *et al.*,

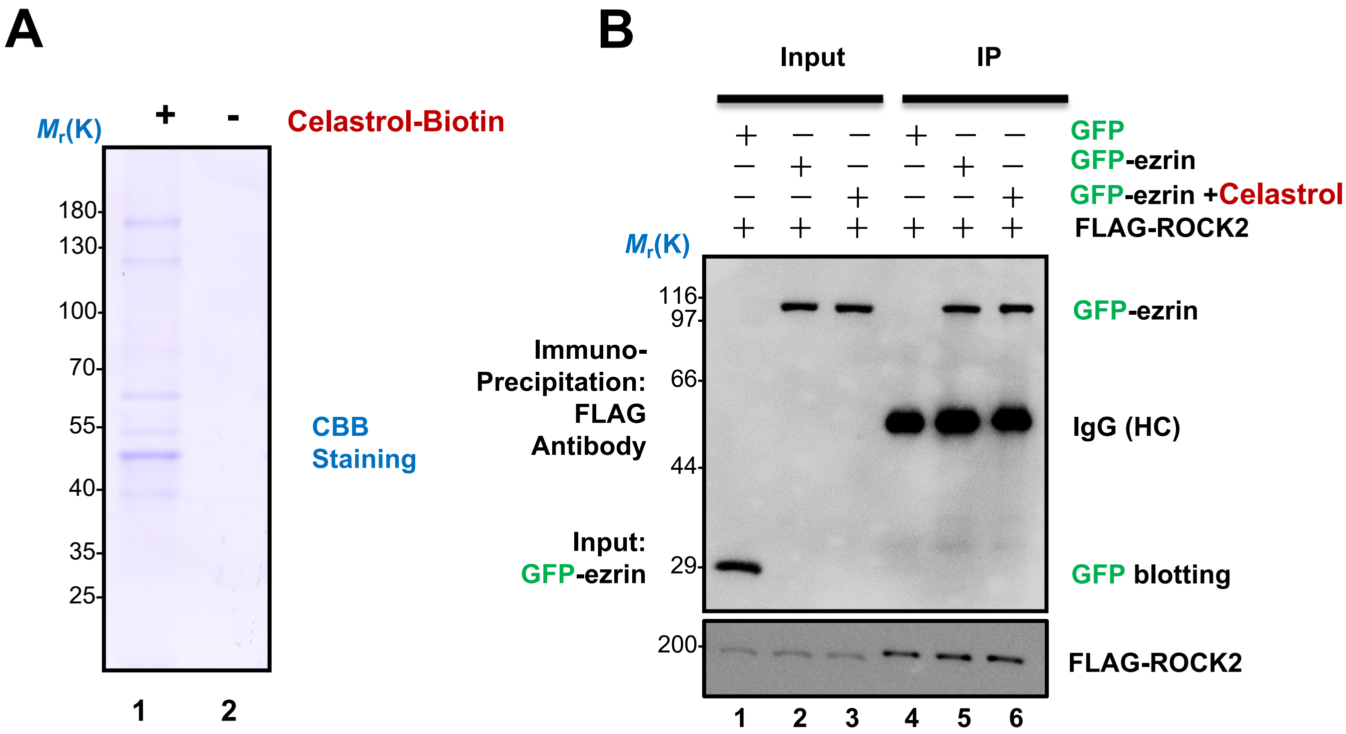

Figure S4

A. The coomassie brilliant blue (CCB) stain result shows the protein pulled down by celastrol-biotin. The MHCC97H cell lysates were treated with 10 μM celastrol-biotin and equal volume of DMSO (-), and then incubated with avidin-beads.

B. Western-blotting result of the co-immunoprecipitation assays. HEK293T cells transfected with Flag-ROCK2 and GFP-ezrin were lysed and incubated with FLAG M2 beads, followed by the western blotting with anti-GFP and anti-FLAG antibodies. The transfected cells were treated with 500 nM celastrol for 6 hours, while the control group were treat with equal volume of DMSO. The cells transfected with Flag-ROCK2 and GFP vector were negative control. Full-length blots are presented in Supplementary Figure S8.

Supplemental Figure 5

Du *et al.*,

A

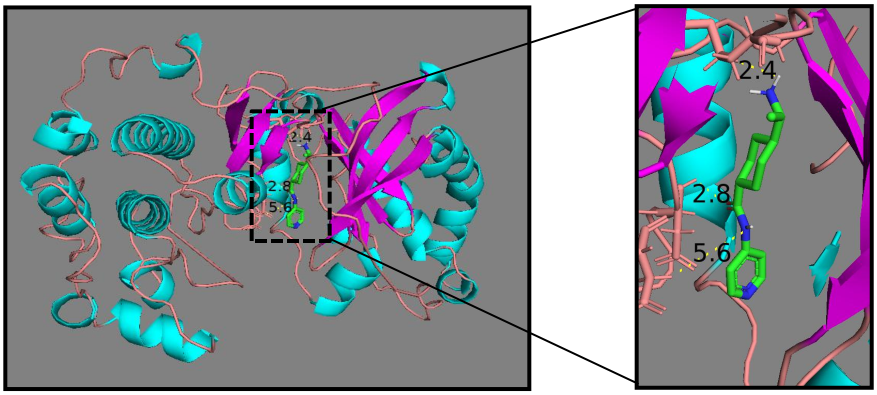

B

Residues	Distance (A^0)
MET-172	2.4
ASP-218	5.6
ASP-232	2.8

Figure S5

A. Cartoon representation of Y27632 (green) docked onto the ROCK2 model. Potential area of Y27632 interacted with the residues of ROCK2 were shown in yellow dotted lines. The numbers represent the distance between each residue and Y27632.

B. The amino acid residues of ROCK2 that formed polar bonds with Y27632 and the distance between each residue and Y27632.

Supplemental Figure 6

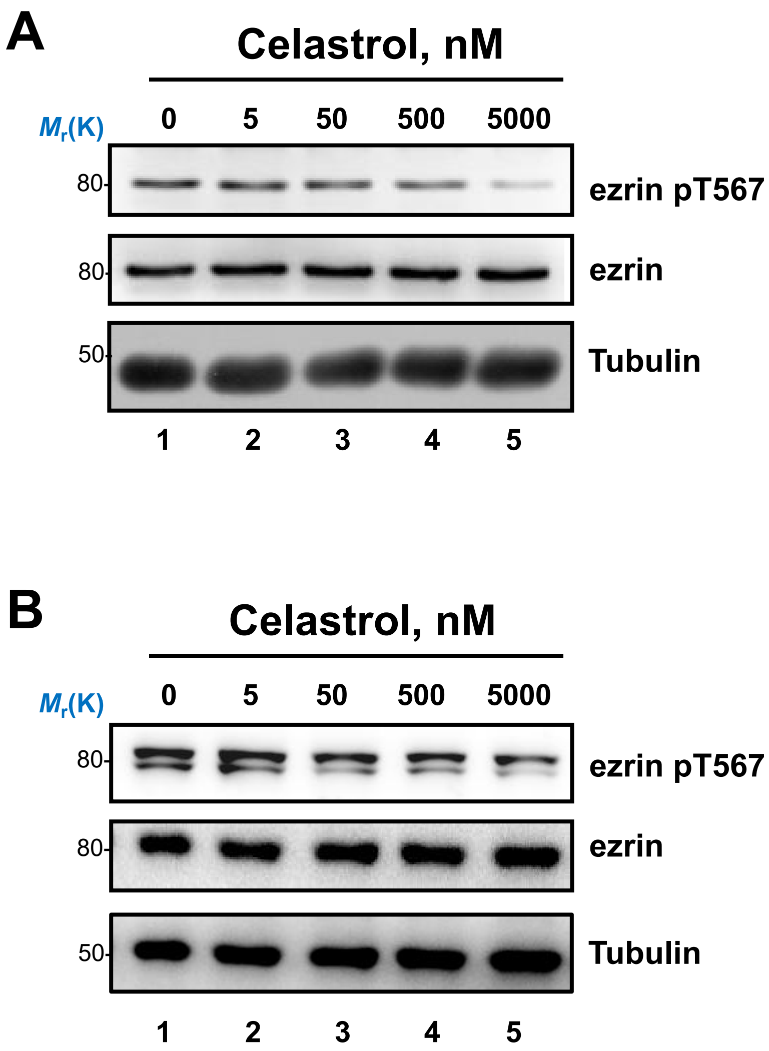

Figure S6

A. Western blotting analyses of ezrin pT567, ezrin and Tubulin, after treating Huh7 cells with different concentration of celastrol for 6h. Full-length blots are presented in Supplementary Figure S8.

B. Western blotting analyses of ezrin pT567, ezrin and Tubulin, after treating HepG2 cells with different concentration of celastrol for 6h. Full-length blots are presented in Supplementary Figure S8.

Supplemental Figure 7

Du *et al.*,

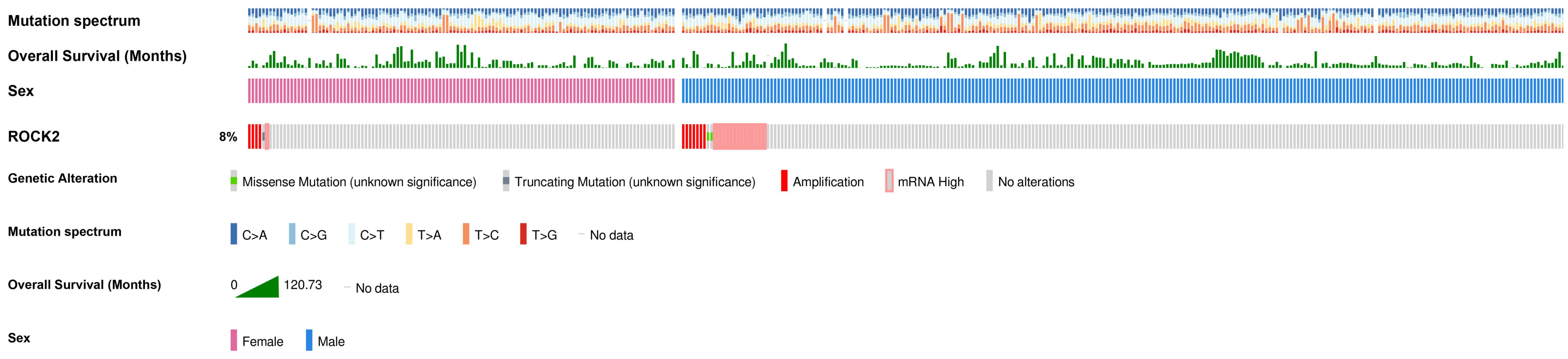

Figure S7
Oncoprint visualization of clinical and genomic features of 442 TCGA hepatocellular carcinoma. Clinical information for this example includes mutation spectrum, overall survival and sex. ROCK2 genomic features are visualized, including mutations, copy number amplifications and mRNA. Individual columns are for individual patients. Patients with the indicated alterations are shown.

Supplemental Figure 8

Du *et al.*,

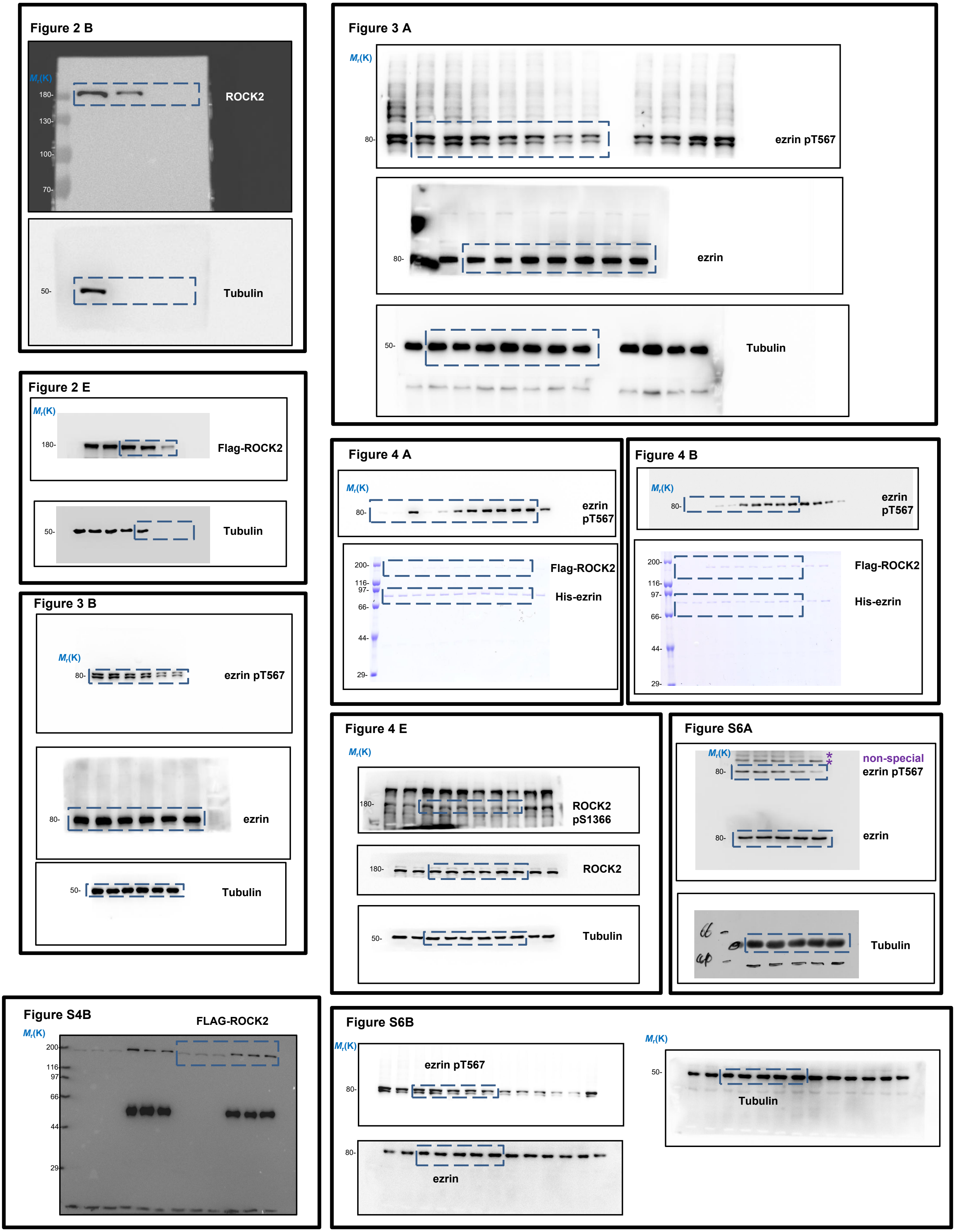

Supplement: Supplementary file 1 — Supplementary Information. [file 41598_2020_68238_MOESM1_ESM.pdf]
